# Screening and Referral for Social Determinants of Health: Maternity Patient and Health Care Team Perspectives

**DOI:** 10.1089/heq.2022.0020

**Published:** 2022-12-05

**Authors:** Kristin P. Tully, Amelia N. Gibson, Marina S. Pearsall, Kelly Umstead, Carolina Gill, Alison M. Stuebe

**Affiliations:** ^1^ Department of Obstetrics and Gynecology, School of Medicine, University of North Carolina at Chapel Hill, Chapel Hill, North Carolina, USA.; ^2^ Collaborative for Maternal and Infant Health, Departments of Obstetrics and Gynecology and Pediatrics, University of North Carolina at Chapel Hill, Chapel Hill, North Carolina, USA.; ^3^ College of Information Studies, University of Maryland, College Park, MD, USA.; ^4^ Department of Maternal and Child Health, Gillings School of Global Public Health, University of North Carolina at Chapel Hill, Chapel Hill, North Carolina, USA.; ^5^ Department of Industrial Design, College of Design, North Carolina State University, Raleigh, North Carolina, USA.

**Keywords:** health risk assessment, minority health, obstetrics, population health, post-partum, pregnancy, screening, referral

## Abstract

**Objective:**

To identify patient and health care team perspectives on screening and referral for Social Determinants of Health (SDoH) in maternity care.

**Methods:**

This human-centered design study was conducted in a prenatal clinic and in the surrounding community of a university teaching hospital in the Southeastern United States. Qualitative data were collected through semistructured interviews and focus groups between March 2019 and February 2020, with findings shared in-person with participants for feedback.

**Results:**

A total of 19 English- and Spanish-speaking patients and 11 health care team members participated. Participants suggested that all patients should be screened as part of integrated health assessments, early in care and periodically, but only when protocols are in place for addressing needs—immediate or ongoing. They expressed concerns that disclosure of SDoH screening data might exacerbate already existing biases, negatively impact care, or be used to harm them. Patients wanted proactive transparency about the purpose of SDoH screening, and to know who would have access to their data, when and how it would be used, and how long it would be stored. Patients expressed concern about confidentiality and stigma, and wanted their health care team to normalize seeking help, and acknowledge that birthing people's circumstances change over time. Patients and health care team responded that patient-provider communication should be respectful, be antiracist, and demonstrate respect for patient autonomy.

**Conclusion:**

Patients and health care team members recommended that SDoH resource information be accessible to all patients regardless of endorsed needs.

## Introduction

Social Determinants of Health (SDoH) are defined by the World Health Organization as “the conditions in which people are born, grow, work, live, and age, and the wider set of forces and systems shaping the conditions of daily life.”^1^ This includes access to food, housing and neighborhood conditions, transportation, personal safety (or exposure to interpersonal violence), community and social support networks, and other contextual threads of daily life. Individual circumstances are shaped by structural, population-level conditions, political and economic policies, power relations, and resource distribution, institutional inequities, and interpersonal relations.^1,2^

Unsurprisingly, inequitable access to SDoH in the United States reflects extensive systemic racial, ethnic, gender, and other discrimination. This translates to inequitable access to health care and drastically different patient experiences within systems of care.^3–7^ Addressing these issues within the context of maternity care offers us the opportunity to improve the health of two generations and may strengthen health care systems for all patient populations.

These problems are not new. Variations in patient care through pre-conception, pregnancy, and post-partum reflect longstanding systemic inequities on multiple levels.^8^ Housing segregation and inequitable community investments create health care and food deserts, increase maternal mortality, and disproportionately harm Native American, non-Hispanic Black, and rural birthing people in many parts of the United States.^9^ For those who do have access, systemic racism, sexism, ableism, and classism may be operationalized as clinical norms, policies, and behaviors.^10^ While individual health care team members have limited control over root causes of systemic inequity, understanding these factors can help inform improvements on individual, interpersonal, institutional, and policy levels.^11–14^ Clinical support typically addresses immediate and near-term individual needs such as food security, diapers, mental health care, and transportation directly in clinical settings and through community partnerships and programs.

SDoH-related programs' potential for protecting and promoting the health of pregnant and birthing people^15^ has been recognized by the American College of Obstetrics and Gynecology (ACOG)^16^ and the American Academy of Pediatrics,^17^ but this work also presents challenges and potential harms. Incorporating SDoH screening and referral into clinical care, maintaining accuracy,^18^ providing meaningful solutions to problems identified, and finding local resources for referrals have all presented limitations to SDoH screening programs.^19,20^ Yet, many SDoH toolkits offer little to no guidance on patient needs for planning and implementation, instead focusing on measures and electronic health record (EHR) integrations. This study explores patient and health care team member perspectives on SDoH in maternity care with a focus on identifying stakeholder recommendations for effective screening and referral.

## Methods

The research project was reviewed by the Biomedical institutional review board of the University of North Carolina at Chapel Hill (No. 18-2811) and determined to be exempt. A human-centered design approach guided the study design and analysis. This methodological framework centers stakeholder perspectives through iterative activities and ongoing analysis with the research team to co-define problems and solutions.^21,22^ Human-centered design has been successfully applied to developing interventions in maternity care.^23,24^

Following informed consent, the study team completed 15 individual interviews, one focus group with 11 maternity health care team members, and one focus group with 4 Spanish-speaking patients between March 2019 and February 2020. The research project included other activities, which are reported elsewhere.^25^

### Setting

The study setting was a prenatal clinic and surrounding community of a university teaching hospital in the Southeastern United States. The prenatal clinic participates in the state's Medicaid Pregnancy Medical Home Program. During the data collection period, SDoH screening for all patients was not part of standard clinical care at the site. A state-level resource portal with local SDoH resources was known to be forthcoming, but not yet available.

### Methodology

Interview guide development was structured to address individual-level screening and referral experiences. The English- and Spanish-language interviews started with affirmation of the research purpose and confidentiality and a description of how participants' responses would be analyzed and shared. Then, question items were about experiences in health care—including what the individuals were asked about in clinical encounters, what sort of information was provided to them, the importance (or not) of health care team members knowing certain aspects of patients' lives, clinical information sharing expectations and preferences, and resource provision.

The interviews closed with determining participant priorities for research team action and offering gratitude. We recruited patients through university e-mail listserv, social media, and a community health center contact sharing information with their clients. The first author (White nonclinician researcher) conducted 8 English-language patient interviews by telephone. In addition, 7 Spanish-language interviews were conducted by a trained, native Spanish-speaking research assistant (Hispanic nonclinician researcher).

Health care participants were recruited through e-mails from the study team, which included a practicing physician from the clinic in which the participants were drawn (White clinician researcher). Health care team members were asked about assessing patient SDoH needs, how the health care professionals learn about resources, their responses to time-sensitive SDoH needs, and how they might build on existing SDoH programs in the clinic setting. In addition, we conducted a focus group with four Spanish-speaking patients. This focus group was added after early analysis of interview data suggested that Spanish-speaking participants may feel more comfortable sharing information in a group format. The focus group was conducted in Spanish (Hispanic nonclinician researcher) and held in a private room in a community health center, with optional on-site childcare provided by the study.

### Participants

To enroll in the study, participants had to be at least 18 years of age at enrollment, be English or Spanish speaking, and have experienced maternity care within the previous year (as a patient or health care professional). In this article, we use the term patient to describe participants who were expectant or recent birthing parents. We use the term health care team for those with medical training and professional staff in the health care setting. Participant roles included clinic managers, front desk clinic staff, pregnancy care managers, social workers, midwives, maternal fetal medicine obstetricians, and nurses. Patient participants were provided with gift cards and health care team members in the focus group were provided lunch.

### Analysis

Early thematic analysis of interviews informed development of the clinical focus group guide and the inclusion of the Spanish-language focus group. Once data collection was complete, we read transcriptions in their entirety and developed an *a priori* framework based on broad topic areas addressed in the data. This framework was used to conduct iterative thematic coding of interview and memo data, and to generate memos related to the open-ended responses. Study investigators initially coded collectively using sticky notes on a dry erase board to visualize content, continuing iterative coding individually using NVivo (version 12, QSR International). Differences in interpretations of themes involved re-reading the responses and investigator discussion to reach consensus.

## Results

### Sample

This analysis includes data from a total of 30 participants: 19 patients and 11 health care. Most patient participants (15/18, 83.3%) reported having public insurance (Medicaid), with 1 insurance status unknown to researchers. Interviews were conducted with 15 patients (3 non-Hispanic White, 3 non-Hispanic Black, 2 Asian, and 7 Hispanic). The health care focus group included 11 individuals (7 non-Hispanic white, 3 non-Hispanic Black, and 1 Hispanic), and the patient focus group included 4 Hispanic individuals.

### Results

Participants suggested that when SDoH resources are available, all patients should be screened as part of integrated health assessments that include SDoH and non-SDoH topics at the same time, first early in care and then periodically. They suggested that screening should not be conducted unless clinics have protocols in place for addressing urgent, immediate needs as well be ready to offer referrals for other high-quality resources. Patients were concerned about bias and stigma, and wanted their health care team to really listen to their concerns, to understand financial and other challenges as normal (and not a cause for mistreatment), and to normalize seeking support. Patients also wanted health care professionals to understand that patient needs and circumstances often change over time.

**FIG. 1.**
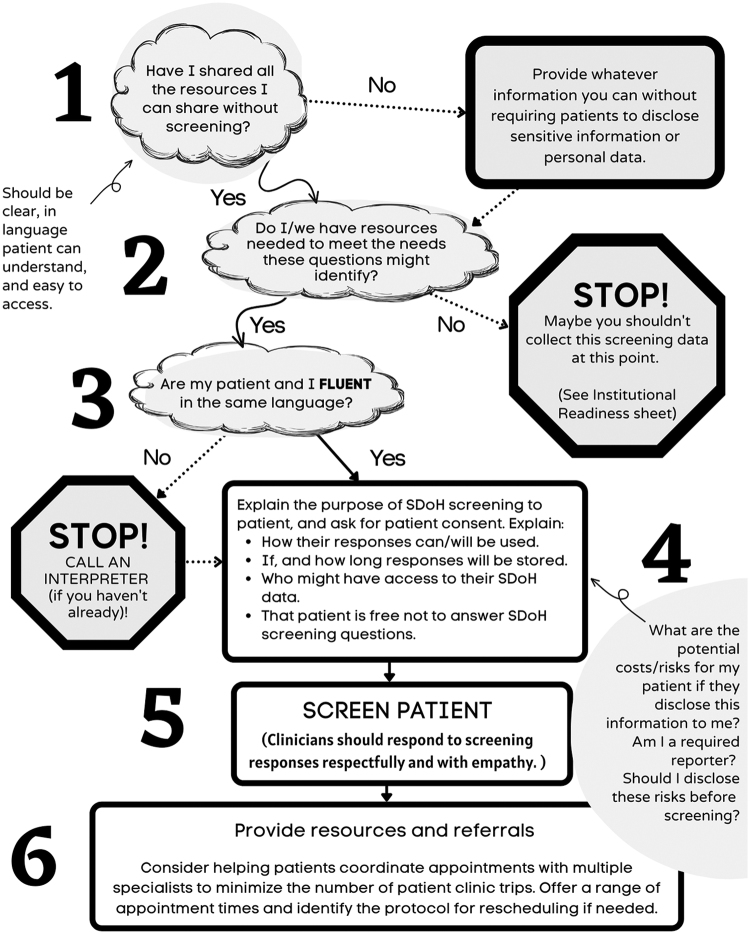
Decision tree for SDoH clinical screening and referral. SDoH, Social Determinants of Health.

Themes across the qualitative data were as follows: SDoH as a necessary part of maternity care, SDoH communication and information exchange as important, yet poorly supported/executed, barriers to disclosure, health care team preparation for SDoH screening and support, leading with resources, and potential clinical improvement strategies. These results are summarized in Table 1. In addition, SDoH clinical workflow considerations from the participant recommendations are addressed in Figure 1. Facilitators and barriers for effective SDoH screening and referral in maternity care are listed in Table 2.

**Table 1.** Themes from Maternity Patient and Health Care Team Participants

Themes	Patients' perspectives	Health care team perspectives or health care systems issues
SDoH as a necessary part of maternity care	The role of the health care team is to “make sure that the patient is doing well.” Patients wanted resources to take the best care of their babies. SDoH care felt like an important part of maintaining a “stable pregnancy” and as part of shared decision making with clinicians	Challenges related to SDoH arise frequently in maternity care, impact patient health, and fuel disparities in outcomes. Not screening and not offering resources can feel akin to “sticking your head in the sand.” With better knowledge of patients' circumstances, health care teams could better address patient priorities and avoid negative labels (e.g., “noncompliant”). Encounters could include validation of patient strengths to build on what is working well. Managing medical complexity with SDoH considerations is especially challenging
SDoH communication and information exchange as important yet poorly supported/executed	Patients should be proactively informed of all available SDoH supports in an organized, accessible format. Health care team members should undergo specific SDoH cultural sensitivity training, and with respect for patients' mental health. Health care professionals should know how to respectfully (and empathetically) screen patients who might be unhappy or uncertain about continuing their pregnancies. SDoH engagement should continue throughout pregnancy “in case their circumstances change,” (e.g., employment loss or lack of paid family leave) or if patients decide they might like “outside help”	Limited visit time. Lack of reimbursement by payers for health care provider SDoH counseling time. The health care team participants felt that SDoH can fall off as a priority in practice. Unaddressed SDoH challenges often manifest later as challenges with fully accessing or utilizing health care (e.g., missed appointments or not filling prescriptions). Lack of knowledge about relevant resources. Information channels about community and hospital programs (e.g., e-mails, flyers, electronic health record) were difficult to manage and often out of date
Barriers to disclosure	Patient concerns about privacy, preserving their agency, and controlling information about their personal lives. Racist/sexist encounters—perception of health care team bias. Perception that a health care team member is withholding information or discourages patient questions. Perception that questions can be unjustifiably invasive. Screening format preferences varied (e.g., paper and pencil or face to face discussion). Lack of information about possible benefits of disclosure—patients wanted to know about available programs, as part of informing their decisions about the possible costs and benefits of disclosing basic needs. Concerns about stigma or other negative consequences of disclosure. Lack of relationship with their health care team and need to build trust. Perceived lack of choice/autonomy in accessing SDoH resources—patients described wanting autonomy in engaging with SDoH resources or not, with follow-up from their health care team about if more information would be helpful, not tracking their use of resources	A Black health care team member discussed a “tale of two stories” with SDoH screening. The individual suggested that patients will offer different responses depending on who is asking. Entry of SDoH screening responses into the EHR was particularly concerning for some patients. Recording and retention of sensitive health information was not in alignment with their interests, unless it is used for direct assistance. Patients also expressed concern about the potential for inaccurate data about them to be retained in the EHR. Some patients conveyed distrust of SDoH screening, citing the possibility of long-term data storage for the health care system's “own purposes.” The patients were generally unsure about whether their SDoH responses would be documented, how long the data would be retained, and whether the information could be shared without their consent. Many of the patients wanted to know upfront how SDoH responses would be used and accessed—particularly if there was any chance that their answers could be used against them. Patients were concerned about targeted surveillance initiated under the auspices of social service provision
Health care team preparation for SDoH screening and support	Patients in the study conveyed desire for their health care team members to undergo sensitivity training to promote more empathetic communication and demonstrations of respect. The patients suggested that appearances can be deceiving and called for attention to “middle class” people, who might be assumed to not have SDoH challenges. For urgent needs, patients suggested clinics have protocols and supplies such as bags of food for immediate response	The health care team members discussed desire to incorporate SDoH into health care services on a deep, meaningful level. SDoH screening and referral processes are challenging from health care team member perspectives. They reported not receiving training on how to communicate on these topics with patients. Additional barriers to supporting people around SDoH from the health care team perspectives were that they were not familiar with the full range of staff and material resources in the clinic and they were unsure of the quality of local community programs. High clinical staff turnover and cross-covering staff who were unfamiliar with clinic protocols were also challenges. Health care team responses were focused on being equipped to care for patients, including who are undocumented or might otherwise be afraid to ask questions. At the same time, the health care professionals articulated the potential for having a “normal bias” about their patients' needs, such as assumptions of food security among those with private insurance
Ideal SDoH engagement leads with resources	Patients reported that when people are dealing with unmet needs, they are “not in the mind state to go research a lot.” Therefore, the consensus from these participants was for clinical provision of clear, actionable information so that people feel and are cared for. Pregnancy intake visits or other early clinical engagement seemed like the most appropriate time to patients for SDoH screening, which they said should be accompanied by clinical review of the responses before or during an appointment. Patient and health care team participants suggested that as part of standard care for each patient, provision of SDoH resources was welcomed. In addition, a patient participant flagged that SDoH resource information being available before the first prenatal care appointment was important. When SDoH needs are endorsed, patients suggested health care team members verbally acknowledge their responses and offer specific resources. Patients also preferred materials be developed as “something they can just grab,” which was similar to health care team considerations of easy ways to provide SDoH information, like a handout. A patient offered that such an approach would also permit individual privacy in familiarizing with programs	Low patient literacy levels were addressed by patients and health care team members in this study and a patient suggested that a phone line to talk through resources would be helpful, in addition to written materials and a website. In addition, the electronic health care portal was offered as a secure place to share information, with health care team members posting resources and patients messaging. A patient shared that “as many different avenues as you can have available, that would be ideal” for SDoH communication and information exchange. Patients addressed wanting their health care team to let them know that if they have any questions or would like additional information at any point, they should feel free to reach out and ask
Potential clinical improvement strategies	A patient shared that the framing of social services or other SDoH supports as key to “co-pilot” their health care. In addition, timing of health care system administrative needs was important. A patient recounted distress at being put in the position of seeing a financial counselor for the cost of an ultrasound scheduled to confirm a miscarriage. An example of helpful engagement from participants was the opportunity for Medicaid transportation to and from appointments, since paying for parking at clinical sites can be stressful and expensive	Health care team members suggested that components of ideal workflow included offering SDoH orientation for new residents and working as a care team for each patient, with routine contact among the health care team to review and coordinate care plans. They further recommended serving as a connector seemed more manageable then being the sole or primary source for SDoH resource recognition and the only communication channel. Group huddles before clinic shifts were recommended so that health care team member names, office locations, and SDoH resource programs were “fresh.” Health care team ideas shared for future SDoH exploration included digital tools such as patient-facing websites or apps (while also providing written materials for those with limited access or technology literacy), texting services, waiting room visual displays, coordination of appointments, community health workers, bilingual clinic volunteers, and patient support groups. The health care team members also suggested implementation of visual cues within clinic encounters (e.g., a colored, laminated card) to safeguard against patients missing out on services

EHR, electronic health record; SDoH, Social Determinants of Health.

**Table 2.** Social Determinants of Health Screening and Referral Facilitators and Barriers from Patients and Health Care Team

Themes	Facilitators	Barriers
Trust among patients and their health care team	Health care systems and health care professionals are accommodating to patient needs and work to earn trust	Institutional structures and health care interactions can cause patient harm and erode trust
Framing screening for patients	Health care teams normalize patient strengths and challenges and affirm that patient needs and circumstances might change	Patients were concerned about how their SDoH information might be used against them or negatively impact their care; Patients may not want to engage around SDoH with clinicians
Screening approaches	Patient and health care team awareness of universal screening, with tool framing and paper and pencil and verbal response options	Patient concern for targeted patient surveillance; Lack of language-concordance
Health care team responses to screening	Health care team engaged with patient screening responses, with validation of strengths and discussion for shared decision making	Limited duration of visits; lack of financial incentives; health care team biases, racism, and patriarchal views. Health care team inability to “go there” due to managing medical complexity
Clinical coordination for referrals	Health care team using visual cues and other tools to signpost the need to address SDoH in clinical encounters; offering warm handoffs	Inadequate clinical knowledge management of resources, including from staff turnover and changing SDoH resources. Limited clinical ability to coordinate patient care plans across health care team members
SDoH resource access for patients	Handouts and websites with information for patients to familiarize with programs in their own time and privately	Differential resource availability to patients based on their insurance payer status, including care management
Patient comprehension	Support for patient comprehension such as a phone line to talk through resources	Health care system structure and resources not adequately described, including in patients' preferred language
Health care team communication with patients	Health care team members proactively communicate that they can offer additional information on resources or patient privacy protection at any point	Lack of health care team training and practice on communicating on SDoH topics with patients. Limited health care team familiarity with SDoH personnel, programs, or policies

Overall, patients positioned their pregnancy experiences within the context of their ongoing lives, with some strengths and challenges persisting and others fluctuating through prenatal and post-partum care. Patients and health care team members in this sample indicated that patient history, feelings of safety, mental health, and other needs are interrelated. Patients had concerns for potential mistreatment or harm because of SDoH disclosure, and those worries influenced their screening responses. They described that patient responses to SDoH screening can reflect the extent to which they feel understood by their health care team and feelings on whether sharing information would translate to help. Some patients described being “treated as if I knew nothing” or having negative reactions from their health care team to their questions, which they attributed to racism.

When clinicians were aware of patient SDoH issues, they said that they would often contact a pregnancy care manager. However, the care manager role was limited in availability to patients with Medicaid. In circumstances in which patient needs were present, but there was no care manager access, the health care team members reported that they did not always know how to respond. They could feel like their “hands are tied” due to lack of familiarity with SDoH resources and visit time constraints. This resulted in patients having to navigate services “on their own.”

## Discussion

This study documented patient and health care team member perspectives on SDoH in maternity care. Participants described clinical SDoH engagement as relevant to improving health. Patients and health care professionals recommended that screening be conducted universally rather than targeting specific populations, that SDoH questions be integrated with other health assessments, and that clinics lead SDoH efforts with resources for patients. They recommended that SDoH resource information be offered in multiple formats to allow patients to browse resources privately and this information should be shared proactively, regardless of needs expressed. The patients desired access to these resources free of judgment and without needing to divulge information that may become a permanent part of their EHR.

In addition, several patients noted wanting to connect with and trust their health care team. For other patients, their focus was on eliminating indifferent, racist, or patriarchal interactions within clinical encounters. Patients and health care team members collectively described the need for health care professionals to demonstrate respect through interpersonal interactions, structure attention to patient priorities, and collaborate for effective care planning.

This qualitative study was intended to examine patient and health care team accounts to identify possible contextual conditions for effective SDoH care. Inclusion of both patients and health care team members in the study is a strength.

In addition, the research team have various disciplinary training (anthropology, industrial design, information sciences, nutrition, public health, and obstetrics), racial-ethnic characteristics (non-Hispanic Black, non-Hispanic White, and Hispanic), native languages (Spanish and English), and experiences as maternity patients and health care team members. The study is limited in that the participants were diverse, but not a representative sample. Furthermore, we recognize that the information shared in research reflects participant trust and their decisions about what is worth sharing, like patients in this study described SDoH engagement in maternity care.

The findings are consistent with a National Academies of Sciences, Engineering, and Medicine (NASEM) report on SDoH implementation.^14^ The NASEM report identified the need to assess SDoH in a manner concordant with a patient's preferred language, literacy, and cultural affiliation and using patient-centered language to communicate how the clinic will use ascertained information to assist the patient. We similarly found that patients want transparency with SDoH screening, to weigh potential costs and benefits of disclosure. The National Academies report also noted that best practices require that an intervention be in place to address identified needs,^14^ which our participants echoed. Furthermore, we heard patients' concerns about documentation of SDoH responses and why storage of these data may disincentivize accurate patient reporting. Patient concerns of how their SDoH information might be used against them should motivate a careful, intentionally nonextractive approach to clinical screening and referral.

Ideally, SDoH screening and referral are built on a shared mental model, with patients and health care team members understanding the purpose, potential risks, and benefits. This collaborative approach may help eliminate stigma, which increases patient stress and restricts their access to health-enabling resources.^26^ The National Association of Community Health Centers offers the Protocol for Responding to and Assessing Patients' Assets, Risks, and Experiences (PRAPARE) for standardized assessment and to inform process.^27^ The PRAPARE toolkit emphasizes empathetic inquiry, including patient-centered support for autonomy/privacy, clear explanations for screening, sharing power by asking about patient priorities, and accounting for stigma. Similarly, the NC Maternal Mental Health MATTERS Toolkit^28^ acknowledges that screening conversations with pregnant and post-partum people can feel daunting to health care professionals.

The MATTERS Toolkit outlines a patient-centered approach, including recommending normalizing statements, explanations of intentions, aligning with patients, and creating open spaces for discussion. MATTERS also recommends communicating institutional privacy/data use policies to patients before screening, for patient reassurance and to inform decisions about how they might prefer to control their personal medical information. Consistent with these tools, our findings suggest that clinical SDoH materials should include written descriptions of the purpose of screening and information about potential future data use.

The first step to effectively address SDoH in clinical care is not screening; results from this study suggest leading with resource information for all patients. Proactively communicating available programs encourages and respects patient autonomy. This is important for all patients and critical for Black, Native, immigrant, disabled, and others who negotiate risks associated with bias and discrimination. Potential harms of disclosure include vulnerability to deportation, family separation, loss of other services, or stigma associated with use of public resources.^29–32^ Therefore, patient self-preservation might take the form of nondisclosure. In addition to transparency of resources, patients' concerns should be allayed by normalizing SDoH supports and building trustworthy, confidential systems that minimize risk and support whole-person care.

**FIG. 2.**
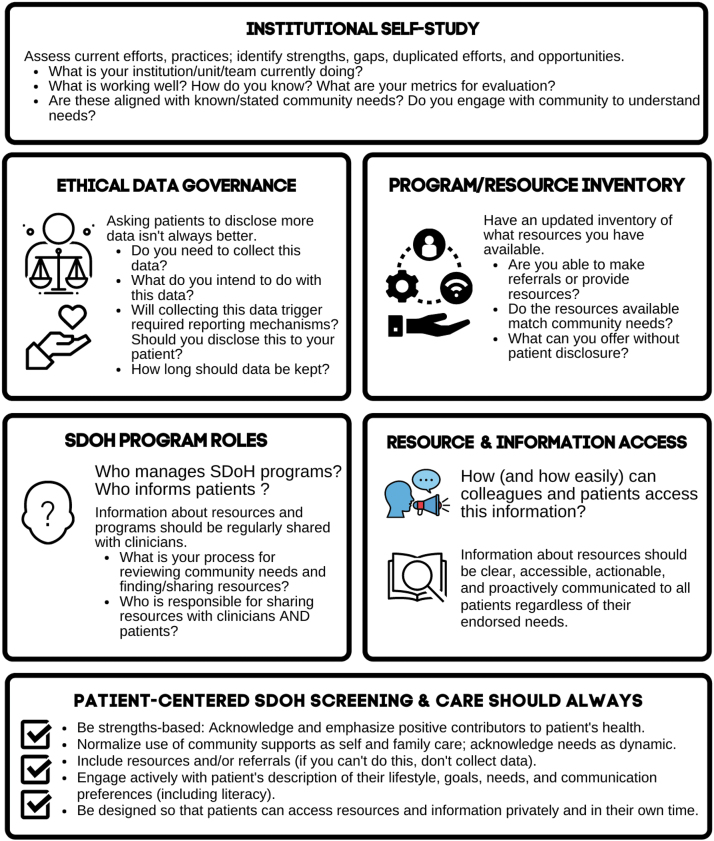
Institutional readiness for SDoH clinical screening and referral.

Patients and health care team members suggested that screening should only occur when support is available and can be accessed. When SDoH screening happens without resource provision or referrals, questions can be perceived as intrusive and harmful, rather than as a tool for identifying support possibilities. To contribute to institutional self-assessment of readiness to address patient needs, we developed Figure 2. This institutional readiness tool outlines components of self-study regarding current efforts, practices, and metrics for evaluation.

SDoH data can potentially facilitate development of tailored care plans for individual patients, inform community and population health management, and provide data for research. Yet, for health care to authentically advance equity and promote health, we might critically assess clinical data governance and move away from defining SDoH success as screening completion. Instead, we can center attention and structural competency around patient-reported metrics of clinic support. ACOG recommends that clinicians foster patient agency across all encounters through compassionate responsiveness.^33^

We similarly suggest trauma-informed^34^ attention—realizing the importance of SDoH interactions, recognizing our capacity to build on patient strengths, and responding without introducing potentially harmful surveillance systems that could both re-traumatize patients and perpetuate negative biases.

That said, changing institutional approaches to SDoH screening and referral comes with opportunity costs, and it is important that patient-centered values guide our priorities as we address competing demands. Integration of SDoH data into EHRs is only important if patients can and should trust us with this information. Prioritizing patient needs and preferences might come at the expense of maintaining complete or permanent records of SDoH.

Our data suggest that in an ideal health care system, patients are free to decide what information to share and what resources to utilize. Responses to screening reflect trust and confidence in the clinic and an honest, uncoerced request for help. If needed, health care team members are properly trained to ask, document, and refer in ways that avoid harming or marginalizing patients, build rapport, and lead to timely provision of accessible, quality resources. Participants in this study echoed the “hidden resources” of health education and community assets identified by Black women in previous research on their experiences with structural racism.^35^

Patients and health care team members in this project described unequal access to resources and opportunity, through haphazard SDoH resource provision as a part of fragmented maternity care. To move closer to the standard of care described by maternity patient and health care team members, clinics need incentives (or at least removal of disincentives), and institutional support to effectively integrate SDoH care into more coordinated systems of practice.

## Conclusions

Effectively addressing patient needs requires broadening the clinical lens beyond the traditional biomedical-centric approach and toward more holistic care (e.g., Refs.^36,37^). Health care team members should be aware of the causes, impacts, and signs of individual needs and stressors related to SDoH, and the implications of structural factors that impact health care on a larger scale. Growth mindsets prepare health care professionals to learn to demonstrate respect in maternity care and address patient needs. As Crear-Perry and colleagues describe,^38^ birth inequities are driven by layers of oppression.

A patient-centered implementation strategy for incorporating SDoH into care for birthing people and their infants and for all patient populations might first consider the ways that assessing SDoH can do harm. These problems include feelings of being targeted, re-traumatized, and surveilled. Good intentions are not synonymous with good care. Health care systems should be accountable for providing patient-centered and useful services, not perpetuating performative or overwhelming approaches to care such as screening without resource provision for any and all endorsed needs. Strategies for continual improvement might include equipping health care teams to be transparent with what patient screening responses might and might not lead to, normalizing stigmatized topics, and preparing individuals to respond with empathy and offer clear information.

SDoH in maternity care occurs in the context of patient encounters that do not always address their priorities,^39^ so this topic is part of the broader story of better aligning services with patient needs and preferences. Human-centered design can center the voices, experiences, and needs of patients and their health care teams to inform and subsequently shift the culture of health and practices. This line of work, including removing financial barriers to quality care, “has the potential to facilitate the identification of interventions and policies that can remediate and eliminate inequities in health across the population.”^38^

## Abbreviations Used

ACOG: American College of Obstetrics and Gynecology
EHR: electronic health record
NASEM: National Academies of Sciences, Engineering, and Medicine
PRAPARE: Protocol for Responding to and Assessing Patients' Assets, Risks, and Experiences
SDoH: Social Determinants of Health
